# The Number of Patients with Acute Myocardial Infarction Decreased and Door-to-Balloon Time Delayed in COVID-19

**DOI:** 10.1155/2021/6673313

**Published:** 2021-03-25

**Authors:** Tianyi Ma, Yuli Huang, Wensheng Li, Jianghua Zhong, Hui Yang, Yilei Zhou, Meijun Li, Wenhao Zhong, Yue Cao, Shijuan Lu, Yunzhao Hu

**Affiliations:** ^1^Department of Cardiology, Shunde Hospital, Southern Medical University, Foshan, China; ^2^Department of Cardiology, Affiliated Haikou Hospital of Xiangya Medical College, Central South University, Haikou, China

## Abstract

**Background:**

At present, COVID-19 is sweeping the world, and all countries are actively responding. During the COVID-19 epidemic, the treatment of patients with acute myocardial infarction (AMI) may be affected.

**Methods:**

We reviewed data of patients with AMI from January 23 to April 23, 2020 (2020), and January 23 to April 23, 2019 (2019), who were admitted to two hospitals from Southern China. We collected clinical characteristics, comorbidities, treatment, prognosis, and key time segments to analyze.

**Results:**

The total number of patients that had been diagnosed with AMI in the two hospitals was 218 in 2020 and 260 in 2019. The number of AMI patients that were admitted to hospitals per day decreased in 2020. The percentage of patients with AMI who refused hospitalization in 2020 was significantly higher than that in 2019 (5.0% vs 1.5%, *p*=0.028). There is no statistical difference in symptoms of the first medical contact (S2FMC) time between 2020 and 2019 (*p*=0.552). Door-to-balloon (D2B) time of ST-elevation myocardial infarction (STEMI) patients who were treated with a primary percutaneous coronary intervention (pPCI) in 2020 was 79 (63.75–105.25) mins, while D2B time in 2019 was 57.5 (41.5–76.5) mins, which was statistically different from the two groups.

**Conclusions:**

COVID-19 had an impact on the number of AMI patients who were admitted to hospitals and the time of treatment. During the COVID-19 epidemic, the number of AMI patients that were admitted to hospitals per day was decreased, while the percentage of AMI patients that refused therapy in these two hospitals increased, and the D2B time of STEMI patients was also delayed.

## 1. Introduction

In China, acute myocardial infarction (AMI) is a major burden on healthcare systems. Shortening total ischemic time is key to the treatment of AMI, and emergency time directly affects the incidence of adverse cardiovascular events. It is important to shorten prehospital emergency time and shorten the time from the first medical contact to the opening of diseased blood vessels in patients with AMI. If patients with acute chest pain can be diagnosed as soon as possible and receive the best treatment plan, especially reperfusion therapy, the mortality of AMI will be reduced and patient outcomes will be improved.

In recent months, COVID-19 was sweeping the world. However, the crisis was showing a particular impact on transportation, diagnostic, and treatment pathways for patients presenting with acute coronary syndrome (ACS) [1]. This particular situation may have a negative impact on the desire for AMI patients to visit the hospital. To understand the treatment of patients with AMI during the COVID-19 epidemic, we collected data of patients with AMI in two hospitals from Southern China for analysis.

## 2. Methods

### 2.1. Study Design and Population

This is a retrospective study. We reviewed data of patients with AMI from January 23 to April 23, 2020 (2020), in Shunde Hospital of Southern Medical University and Affiliated Haikou Hospital to Xiangya Medical College of Central South University. We used the fourth universal definition to diagnose AMI. The current fourth universal definition defines myocardial infarction (MI) as the presence of acute myocardial injury. In evidence of acute myocardial infarction, cardiac troponin valued above the 99th percentile of the upper reference limit (URL) and symptoms, electrocardiogram (ECG), imaging changes, or angiographic findings of local ischemia [2]. AMI included ST-elevation myocardial infarction (STEMI) and non-ST-elevation myocardial infarction (NSTEMI). The control group was patients with AMI who were admitted to the same period in 2019 (January 23 to April 23, 2019). We excluded patients with AMI whose onset was more than 7 days at the time of the first visit, patients with onset in the hospital, and patients who underwent percutaneous coronary intervention (PCI) after thrombolytic therapy. The study complied with the principles of the Declaration of Helsinki and was approved by the local ethics committee (no. 2020070).

### 2.2. Data Collection and Analysis

We collected demographic information (age and gender), history of previous coronary heart disease, comorbidities (hypertension, diabetes mellitus, ischemic stroke, and hyperlipidemia), type of myocardial infarction, length of hospitalization, death during hospitalization, left ventricular ejection fraction (LVEF) in cardiac ultrasound, and the treatment. We also collected two key time segments, including the time from the onset of symptoms to the first medical contact (S2FMC) and the time of patient's arrival at hospital door-to-balloon (D2B). We compared the basic clinical characteristics and S2FMC time of these two groups of patients, and we compared D2B time between STEMI patients that presented to the clinic within 12 hours of the onset of symptoms and underwent primary percutaneous coronary intervention (pPCI).

### 2.3. Statistical Analysis

All statistical analyses were performed with SPSS version 23.0. Data were expressed as a proportion (%) for categorical variables. The Kolmogorov–Smirnov test was used to identify whether the data was normally distributed. Normally distributed measures were expressed as (means ± SD) and nonnormally distributed measures were expressed as median (M) and quartiles (Q1–Q3). The nonparametric test (Mann–Whitney U) was used for measurement data, and the t-test or *χ*^2^ test was used for counting data. When *p*-value was less than 0.05, the difference was considered significant.

## 3. Results

Clinical characteristics of AMI inpatients are shown in Table 1. Except for hypertension, these two groups are not statistically different in terms of other information, including gender, age, history of diabetes, history of coronary artery disease, history of myocardial infarction, history of hyperlipidemia, and history of ischemic stroke.

The total number of patients with AMI who were admitted in these two hospitals from January 23 to April 23 in 2020 was 218, including 207 inpatients and 11 patients refusing hospitalization in these two hospitals. From January 23 to April 23, 2019 (2019), there were 260 patients with AMI, including 256 patients that were hospitalized and 4 patients that refused hospitalization in these two hospitals. In 2019, 256 AMI patients included 160 patients with STEMI and 96 patients with NSTEMI. In 2020, 207 AMI patients included 123 patients with STEMI and 84 patients with NSTEMI. The number of AMI patients that were admitted to hospitals per day and the number of STEMI patients that were admitted to hospitals per day decreased in 2020. The percentage of patients with AMI who refused hospitalization in the two hospitals in 2020 was significantly higher than in 2019.

As shown in Table 2, S2FMC time of AMI inpatients in 2020 was 251 (80–1080) mins, S2FMC time in 2019 was 309 (84.25–1267.50) mins, and there was no statistical difference between these two groups. Compared with 2019, LVEF, in-hospital mortality, and hospitalization length were of no statistical difference in 2020. The percentage of patients with STEMI who were admitted within 12 hours of symptom onset was not statistically different from the two groups. The number of patients with conservative therapy, the number of patients with thrombolytic therapy, and the number of patients with invasive treatment (including PCI, aspiration thrombectomy, and balloon dilatation) between 2020 and 2019 were not statistically different.

The percentage of patients with STEMI who were admitted within 12 hours of symptom onset and underwent pPCI was not statistically different from the two groups (85.7% vs 82.6%, *p*=0.546). However, D2B time of STEMI patients treated within 12 hours of symptom onset in 2020 was 79 (63.75–105.25) mins, longer than 57.5 (41.5–76.5) mins in 2019 (*p* ≤ 0.01). There were no statistical differences in LVEF, in-hospital mortality, hospitalization length, and involved vessels between 2020 and 2019 (Table 3).

## 4. Discussion

Globally, AMI was characterized by acute onset and high mortality [3, 4]. Patients with AMI need timely medical treatment. In order to reduce the morbidity and mortality of AMI, it is essential that the time of myocardial ischemia must be extremely short and the resumption of blood to the myocardium should be as soon as possible. Time segments such as S2FMC time and D2B time were critical for AMI patients. Recently, there is an unprecedented challenge for China's healthcare system by the COVID-19 pandemic due to the SARS-CoV-2 [5, 6]. The government has taken a lot of measures to limit the spreading of infection [7]. Despite the COVID-19 epidemic, patients with the cardiovascular disease still require urgent medical attention to avoid the detrimental effects of delays in diagnosis and treatment [8]. Meanwhile, COVID-19 patients could be accompanied by myocardial damage and thrombosis. Mechanisms involved may include direct viral myocardial injury, microvascular injury, stress cardiomyopathy, systemic hyperinflammation, and excessive coagulation activation and hyperfibrinolysis. Therefore, SARS-CoV-2 may lead to a higher risk of ACS [9–11]. Another recent study also pointed out the link between COVID-19 and cardiovascular risk factors. Although epidemiological evidence suggested that anti-inflammatory and immunomodulatory properties of statins may reduce the risk of infections and infection-related complications, this study showed that COVID-19 severity is not mitigated by cardioprotective medical treatment [12].

Previous studies have demonstrated that the incidence and mortality of AMI in China were increasing in recent years [13, 14]. However, in the present article, we provide evidence of a significant reduction in AMI admissions after the onset of COVID-19. The total number of patients with STEMI and patients with NSTEMI both decreased in 2020. We also found that the number of AMI patients and STEMI patients per day reduced in 2020. Several studies around the world also have reported decreases in ACS cases admitted during the COVID-19 pandemic era [8, 15–17]. Although our study found that the number of patients that had been diagnosed with AMI in 2020 was reduced compared with 2019, the number of patients that had been diagnosed with AMI but refused to be hospitalized in 2020 was significantly higher than in the same period in 2019. Some patients might refuse to be hospitalized or even refuse to visit a doctor because they were worried about being infected with COVID-19 during hospitalization. However, AMI is a cardiovascular disease requiring hospital treatment.

Comparing S2FMC time in 2019 and 2020, our study found that there was no statistical difference between these data. From January 2020 to April 2020, at the peak of the COVID-19 epidemic in China, many measures have been taken to prevent the spread of infection by our governments. Patients would be extremely hesitant to decide whether or not to go to the hospital. During COVID-19, people would be too afraid of getting infected to seek medical treatment. On the other side, it has been demonstrated that patients know little about AMI symptoms [18]. However, the S2FMC time of patients with AMI has not been delayed due to the COVID-19 epidemic in 2020. This might be related to the establishment of Chest Pain Center (CPC) in Chinese hospitals in recent years. The development of CPCs in China was initiated in 2010, and the headquarters of China Chest Pain Centers was officially established in July 2016 to coordinate social resources and promote rapid development of CPCs [19]. Shorting S2FMC time could reduce short- and long-term mortality and the incidence of major adverse cardiovascular and cerebrovascular events (MACCE) of patients with STEMI [20].

The guidelines clearly indicated that primary percutaneous coronary intervention (pPCI) is an important treatment for STEMI patients within indicated time frames [21]. Reperfusion therapy is still required for patients whose myocardial ischemia time is less than 12 hours and a persistently elevated ST-segment with at least two consecutive ECG leads to persistent ischemic symptoms [21]. D2B time is strongly associated with the morbidity and mortality of patients with STEMI [22]. In this study, the D2B time for the 2020 group was significantly longer than the D2B time for 2019 group. In previous STEMI patient emergencies, the patients with indications for emergency intervention would be directly transferred to the cardiac catheterization laboratory. Some AMI patients were seen via the network before they arrived at the PCI hospital. Prehospital emergency personnel or community hospital doctors transmitted the patient's data to the PCI hospital via the network. Once the patients were diagnosed with STEMI and still need pPCI treatment, they would skip the emergency department visit and arrive directly at the catheterization room for urgent interventional treatment. At present, because there is no rapid diagnosis method of COVID-19, all STEMI patients need to screen for SARS-CoV-2 through medical history collection, body temperature, electrocardiogram, chest CT, and blood sample collection [23]. Emergency interventions will be performed after the initial exclusion of COVID-19. These reasons lead to the prolonged D2B time.

Although D2B time was extended, there was no difference in mortality and hospitalization length of AMI patients between these two periods. There also was no difference in mortality and hospitalization length of STEMI patients and NSTEMI patients between these two periods. Several reasons could explain this situation. First, a 90 min target for D2B time is generally recommended in STEMI patients' care [22]. Although D2B time was delayed, it still <90 min in 2020. Another study of 44 hospitals in England reported that no differences were observed for in-hospital death despite a longer D2B time in 2020 [24]. Moreover, in these two periods, there was no difference in the rate of patients receiving coronary interventional treatment, the rate of patients receiving conservative treatment, and the involved vessels of AMI patients. Furthermore, COVID-19 epidemic requires a lot of medical resources; however, our hospitals still ensured that there were sufficient medical resources and medical personnel to treat AMI patients. At that time, the medical team made full use of the Internet to transmit patient clinical information and made a diagnosis as early as possible.

Cardiac troponin (cTn) is usually used to diagnose AMI, but it is regrettable that we did not collect data of cTn in this study. Compared with the baseline data from 2019, the probability of AMI patients with previous coronary heart disease remained unchanged in 2020. Previous study reported that patients with chronic coronary syndromes (CCS) and COVID-19 have higher cTn values than patients without CCS. Therefore, relying on cTn for the diagnosis of NSTEMI in COVID-19 may determine obvious diagnostic bias in some patients, especially in patients with CCS [25].

In order to shorten the duration of the myocardial ischemia, some measures should be taken. Firstly, the CPCs should popularize knowledge of acute chest pain to the public, so that people can understand myocardial infarction better and raise awareness of medical treatment of chest pain. Secondly, we also need to optimize the process of visiting patients with chest pain during the pandemic of infectious diseases; therefore, patients can quickly access first aid for optimal treatment. Thirdly, a method of rapid detection of SARS-CoV-2 needs to be developed to reduce pPCI preoperative evaluation time. Fourthly, hospitals need to strengthen the prevention and control of nosocomial infections to provide patients with a safer medical environment.

## 5. Limitations

Our study has the following limitations. First, our study was a retrospective study, so there might be some bias in the selection of samples. Second, the two hospitals included in the study were located in cities in southern China, which may have geographical limitations. Third, the number of our research samples was small, and the observation time for mortality and LVEFs relatively was short, which may not reflect the long-term prognosis of patients. Fourth, we did not collect data concerning arrhythmias in COVID-19 patients and ischemic substrate, that showed to be related [26]. Last, troponin values were not measured at the same time point and are not reported in our analysis.

## 6. Conclusions

COVID-19 had an impact on the number of AMI patients who were admitted to hospitals and the time of treatment. During the COVID-19 epidemic, the number of AMI patients that were admitted to hospitals per day was decreased, while the percentage of AMI patients that refused therapy in these two hospitals increased, and the D2B time of STEMI patients was also delayed.

## Figures and Tables

**Table 1:** Clinical characteristics of AMI inpatients.

	2019 (*n* = 256)	2020 (*n* = 207)	*p*
Mean age, *y* (SD)	63.95 ± 13.92	62.69 ± 13.37	0.327
Males (%)	79	73	0.134
Coronary heart disease (%)	14.8	18.4	0.310
Ever-myocardial infarction (%)	8.6	7.2	0.595
Hypertension (%)	53.5	43	0.024
Diabetes mellitus (%)	24.6	18.8	0.136
Hyperlipidemia (%)	5.1	7.7	0.242
Ischemic stroke (%)	18.4	9.7	0.094
AMI patients per day, *N* (Q1–Q3)	3 (2–4)	2 (1–3)	0.011
STEMI (%)	62.5	59.4	0.499
STEMI per day, *N* (Q1–Q3)	2 (1-2)	1 (1-2)	0.038
NSTEMI per day, *N* (Q1–Q3)	1 (0-1)	1 (0–1.75)	0.318
AMI patients refused therapy in the hospital (%)	5.0	1.5	0.028

AMI: acute myocardial infarction; STEMI: ST-elevation myocardial infarction; NSTEMI: non-ST-elevation myocardial infarction.

**Table 2:** Time segment and prognosis of AMI inpatients.

	2019 (*n* = 256)	2020 (*n* = 207)	*p*
S2FMC time (minutes)	309 (84.25–1267.50)	251 (80–1080)	0.552
Conservative therapy (*N*)	50	34	0.389
Thrombolytic therapy (*N*)	1	2	0.443
Invasive treatment (*N*)	205	171	0.488
LVEF	62 (52.5–67.5)	62 (54.5–67.0)	0.826
In-hospital mortality (%)	3.9	3.4	0.765
STEMI in-hospital mortality (%)	3.9	3.3	0.822
NSTEMI in-hospital mortality (%)	3.9	3.6	0.941
Hospitalization length (days)	7 (5–9)	7 (5–8.25)	0.410
STEMI hospitalization length (days)	7 (5.25–9)	6 (5–8)	0.122
NSTEMI hospitalization length (days)	7 (5–9)	7 (5–9)	0.903
Patients admitted within 12 hours of onset (%)	73.4	68.1	0.335

AMI: acute myocardial infarction; S2FMC: symptoms to the first medical contact; STEMI: ST-elevation myocardial infarction; NSTEMI: non-ST-elevation myocardial infarction; LVEF: left ventricular ejection fraction; *N*: number.

**Table 3:** D2B time and prognosis of STEMI patients receiving pPCI.

	2019 (*n* = 90)	2020 (*n* = 78)	*p*
D2B time (minute)	57.5 (41.5–76.5)	79 (63.75–105.25)	≤0.01
In-hospital mortality (%)	5.5	2.5	0.324
LVEF	62.19 ± 8.584	59.67 ± 10.099	0.095
Hospitalization length (day)	7 (5–8)	6 (5–8)	0.256
involved vessels
Left anterior descending	48	43	0.816
Left circumflex	3	3	0.858
Right coronary artery	39	32	0.763

STEMI: ST-elevation myocardial infarction; pPCI: primary percutaneous coronary intervention; D2B: door-to-balloon; LVEF: left ventricular ejection fraction.

## Data Availability

The data used to support the findings of this study are available from the corresponding author upon request.
